# Data Set and Benchmark (MedGPTEval) to Evaluate Responses From Large Language Models in Medicine: Evaluation Development and Validation

**DOI:** 10.2196/57674

**Published:** 2024-06-28

**Authors:** Jie Xu, Lu Lu, Xinwei Peng, Jiali Pang, Jinru Ding, Lingrui Yang, Huan Song, Kang Li, Xin Sun, Shaoting Zhang

**Affiliations:** 1 Shanghai Artificial Intelligence Laboratory, OpenMedLab, Shanghai, China; 2 Clinical Research and Innovation Unit, Xinhua Hospital Affiliated to Shanghai Jiao Tong University School of Medicine, Shanghai, China; 3 West China Biomedical Big Data Center, West China Hospital, Sichuan University, Chengdu, China; 4 Med-X Center for Informatics, Sichuan University, Chengdu, China

**Keywords:** ChatGPT, LLM, assessment, data set, benchmark, medicine

## Abstract

**Background:**

Large language models (LLMs) have achieved great progress in natural language processing tasks and demonstrated the potential for use in clinical applications. Despite their capabilities, LLMs in the medical domain are prone to generating hallucinations (not fully reliable responses). Hallucinations in LLMs’ responses create substantial risks, potentially threatening patients’ physical safety. Thus, to perceive and prevent this safety risk, it is essential to evaluate LLMs in the medical domain and build a systematic evaluation.

**Objective:**

We developed a comprehensive evaluation system, MedGPTEval, composed of criteria, medical data sets in Chinese, and publicly available benchmarks.

**Methods:**

First, a set of evaluation criteria was designed based on a comprehensive literature review. Second, existing candidate criteria were optimized by using a Delphi method with 5 experts in medicine and engineering. Third, 3 clinical experts designed medical data sets to interact with LLMs. Finally, benchmarking experiments were conducted on the data sets. The responses generated by chatbots based on LLMs were recorded for blind evaluations by 5 licensed medical experts. The evaluation criteria that were obtained covered medical professional capabilities, social comprehensive capabilities, contextual capabilities, and computational robustness, with 16 detailed indicators. The medical data sets include 27 medical dialogues and 7 case reports in Chinese. Three chatbots were evaluated: ChatGPT by OpenAI; ERNIE Bot by Baidu, Inc; and Doctor PuJiang (Dr PJ) by Shanghai Artificial Intelligence Laboratory.

**Results:**

Dr PJ outperformed ChatGPT and ERNIE Bot in the multiple-turn medical dialogues and case report scenarios. Dr PJ also outperformed ChatGPT in the semantic consistency rate and complete error rate category, indicating better robustness. However, Dr PJ had slightly lower scores in medical professional capabilities compared with ChatGPT in the multiple-turn dialogue scenario.

**Conclusions:**

MedGPTEval provides comprehensive criteria to evaluate chatbots by LLMs in the medical domain, open-source data sets, and benchmarks assessing 3 LLMs. Experimental results demonstrate that Dr PJ outperforms ChatGPT and ERNIE Bot in social and professional contexts. Therefore, such an assessment system can be easily adopted by researchers in this community to augment an open-source data set.

## Introduction

The development of large language models (LLMs) has revolutionized natural language processing, raising significant interest in LLMs as a solution for complex tasks such as instruction execution and elaborate question-answering in various domains [1]. Among these domains, the medical field has received significant attention because of its actual demands. Recently, progress has been achieved in medical education [2], patient care management [3], medical exams [4], and other medical applications.

Despite their capabilities, LLMs are prone to generating hallucinations (not fully reliable responses) [5,6]. Hallucinations in LLMs’ responses create substantial risks, potentially threatening patient’s physical safety and leading to serious medical malpractice. Thus, to perceive and prevent this safety risk, we must conduct an exhaustive evaluation of LLMs in the medical domain and build a systematic evaluation.

However, conducting an exhaustive evaluation for LLMs is not trivial. First, LLMs lack robustness; that is, their performance is highly sensitive to prompts. White et al [7] showed that a meticulously crafted and thoroughly tested prompt could greatly improve performance and produce superior results. Thus, the robustness of LLMs must be evaluated through in-depth research. Second, the evaluation criteria of LLMs are critical. Recent evaluations have been mainly based on automatic metrics [8-10] (eg, bilingual evaluation understudy, Recall-Oriented Understudy for Gisting Evaluation, and accuracy) in popular applications such as machine translation and text summarization. Despite their high efficiency, these automatic metrics are insufficient for using LLMs in real-world medical scenarios. Other factors such as the logical coherence of responses, social characteristics like tone, and the ability to understand contextual information are essential influential factors [6,11-17].

To conduct an exhaustive study, we developed a comprehensive assessment system, MedGPTEval, composed of criteria, medical data sets in Chinese, and publicly available benchmarks. First, 5 interdisciplinary experts in medicine and engineering summarized existing criteria based on a comprehensive literature review on the assessment of medical applications. The experts have rich research experience in artificial intelligence (AI) or big data, but specific subdisciplines and majors may vary, including AI and health care management, AI and clinical medicine, AI and medical imaging, clinical medicine and big data, AI, medical imaging, and computer vision. Second, these candidate criteria were optimized using a Delphi method. In the realms of health care [18,19] and the foresight of interdisciplinary future-built environments [20], the Delphi method has emerged as an efficacious instrument for amalgamating the insights of experts across diverse domains, fostering consensus, and refining standards. This approach serves to harmonize the interests of all pivotal stakeholders, thereby amplifying the efficacy and transparency of value-based outcomes [19]. The obtained evaluation criteria cover medical professional capabilities, social comprehensive capabilities, contextual capabilities, and computational robustness, with 16 detailed indicators. Third, 3 clinical experts designed medical data sets to interact with LLMs, including 27 medical dialogues and 7 case reports in Chinese. The case data set is adapted and constructed based on real clinical cases. We have adopted multiple rounds of internal review and expert review processes, and have conducted verification consistent with actual clinical scenarios to ensure the accuracy and practicality of the data. Finally, benchmarking experiments were conducted on the data sets. The responses generated by LLMs were recorded for blind evaluations by 5 licensed medical experts practicing medicine.

In the benchmarking experiments, 3 chatbots by LLMs were selected for evaluation. First, ChatGPT, an LLM created by OpenAI, has gained global popularity owing to its exceptional language capabilities [2]. However, ChatGPT has not been specifically trained for the medical domain [21]. Second, ERNIE Bot is an LLM developed by Baidu, Inc, a Chinese computer technology company [22]. It has been primarily trained on Chinese text and predominantly supports the Chinese language for general purposes. Third, Doctor PuJiang (Dr PJ) is an LLM created by the medical research group of the Shanghai Artificial Intelligence Laboratory. Dr PJ has been trained based on massive Chinese medical corpora and supports various application scenarios, such as diagnosis, triage, and medical question-answering. Note that ChatGPT and ERNIE Bot are general-purpose conversational AI systems, while Dr PJ is an LLM fine-tuned specifically for medical use. To promote research on the evaluation of medical LLMs, we conducted benchmarking experiments on the proposed medical data sets in Chinese. Experimental results show that Dr PJ outperformed ChatGPT and ERNIE Bot in both the multi-turn medical dialogues (scores of 13.95 vs 13.41 vs 12.56 out of 16) and the case report scenarios (scores of 10.14 vs 8.71 vs 8.0 out of 13).

The scale of the data set remains limited. We urge researchers in this community to join this open project via email (xujie@pjlab.org.cn). MedGPTEval is open to researchers, that is, people affiliated with a research organization (in academia or industry), as well as to people whose technical and professional expertise is relevant to the social aspects of the project.

The contribution of this work is 2-fold:

1. By conducting a thorough study of LLMs used in the medical context and collaborating with domain experts, we established comprehensive evaluation criteria to assess the medical responses of LLMs.
2. Based on the criteria, we released a set of open-source data sets for the evaluation of medical responses in Chinese and conducted benchmark experiments on 3 chatbots, including ChatGPT.

## Methods

### Evaluation Criteria

The evaluation criteria for assessing the LLMs were summarized by a thorough literature review. The evaluation criteria were then optimized using the Delphi method [23]. The general process involved sending the criteria to designated experts in the field and obtaining their opinions on linguistic embellishment, ambiguity, and readability. After generalizing and corrections, we provided anonymous feedback to each expert. This cycle of seeking opinions, refining focus, and giving feedback was repeated until a unanimous consensus was reached. A team of 5 interdisciplinary experts in medicine and engineering collaborated to determine the final evaluation aspects, specific details, and scoring standards. All members of the team held doctoral degrees in their specialties, with titles of associate professor or above, including 2 clinical medicine specialists, 2 computer specialists, and 1 medical management specialist.

### Medical Data Sets in Chinese

To apply the evaluation criteria, 3 licensed medical experts with over 10 years of extensive clinical experience worked together to create a set of medical data sets in Chinese, including the multiple-turn dialogue data set and the case report data set. The case report data set necessitated a singular round of questioning and encompasses an elaborate medical record of the patient, including age, gender, medical history (personal and familial), symptoms, medication history, and other relevant information. In addition, the medical problem consulted had to be clearly described. In contrast, the data set with multiple-turn dialogue was derived through an iterative process comprising four rounds. The initial round was initiated with the patient’s symptoms, followed by supplementary descriptions of medication, examination, or other symptom-related queries. The data set with multiple-turn dialogue required careful consideration to assess contextual relevance.

### Benchmark

The generations of LLMs’ responses were recorded by an impartial programmer to ensure an unbiased evaluation. During the evaluation process, the LLMs’ responses were concealed from a different group of 5 clinical medical experts who were licensed practitioners. They have similar years of clinical experience, and we have unified training on assessment processes and criteria to account for the impact of differences in clinical practice on the assessment process. The clinical fundamental response performances of 3 LLMs (ChatGPT, ERNIE Bot, and Dr PJ) were then compared based on the assessment criteria outlined above and on the proposed medical data sets. The data sets proposed by 5 clinical medical experts based on actual clinical experience and clinical confusion, and determined through peer review and discussion were used to evaluate the medical and social capabilities of the LLMs, while the multiple-turn dialogue data set was used to additionally assess their contextual abilities. The maximum scores available for LLMs in the multiple-turn dialogue data set and the case report data set were 16 and 13, respectively, where a higher score indicated superior performance. Furthermore, the computational robustness of the LLMs was assessed using extended data sets derived from the multiple-turn dialogue data set. Lastly, a subset of the case reports was randomly selected and comprehensively reviewed by five medical experts. The benchmark assessment methods are summarized in Table 1.

**Table 1.** Summary of benchmark assessment.

Data sets and assessment aspects		Assessment approaches
**Medical dialogue**		
	Medical professional capabilities, social comprehensive capabilities, contextual capabilities	Maximum score of 16
	Computational robustness	Percentage
**Case report**		
	Medical professional capabilities, social comprehensive capabilities	Maximum score of 13
	Computational robustness	Percentage
	Comprehensive review	Comments

### Ethical Considerations

This study does not include human participants (ie, no human subject experimentation or intervention was conducted) and does not require institutional review board approval.

## Results

### Comprehensive Assessment Criteria

The draft evaluation criteria for assessing the LLMs were summarized by a thorough literature review [6,7,11-14,16,17,24] from 4 aspects: medical professional capabilities, social comprehensive capabilities, contextual capabilities, and computational robustness. All 5 interdisciplinary experts made suggestions for fine-tuning the assessment method, and they reached a consensus using the Delphi method to make it more scientifically rigorous and easier to read [23].

#### Medical Professional Capabilities

The professional comprehensive capabilities of LLMs’ answers were evaluated using 6 indicators [7,12,17]: (1) accuracy, requiring that there are no medical errors in the answers and that the answers do not provide any harmful information to patients (accuracy can also include the evaluation of safety); (2) informativeness, where a 3-point Likert scale was used to evaluate the informativeness of the answers (0: incomplete; 1: adequate; 2: comprehensive); (3) expansiveness, meaning that the answers contain useful information besides the medical knowledge included in the question; (4) logic, with a 3-point Likert scale (0: the answer is irrelevant to the topic; 1: off topic, the answer does not directly address the topic but is still relevant; 2: on topic, the answer addresses the topic directly and positively); (5) prohibitiveness, where the LLMs correctly identify medical vocabulary or prohibited vocabulary; and (6) sensitivity, ensuring that LLMs’ answers do not contain any politically sensitive expressions. Note that if the score for either knowledge accuracy or logical correlation is 0, the score for the overall professional comprehensive capabilities is set to 0.

#### Social Comprehensive Capabilities

We conducted an overall evaluation of the social comprehensive performances using 4 indicators [6,11,12,14]: (1) comprehension, where a binary scale is used to evaluate the readability of the answers (0: awkward sounding—all answers are professional and not explanatory; 1: understandable—intuitive and easy to understand); (2) tone, which pertains to the appropriate use of mood/tone in the generated responses by the LLMs, including the use of mood particles, symbols, emotional rhythm, and emotional intensity; (3) empathy, where the accuracy of the scenario analysis is considered, including emotional understanding and reasoning; and (4) social decorum, using a 3-point Likert scale to evaluate the social decorum (0: rude–not matching any friendly social keywords or displaying malicious language attacks; 1: general—matching 1-2 keywords; 2: graceful—matching 3 or more keywords).

#### Contextual Capabilities

Three indicators were used to access the contextual capabilities [13,24] only in the multiple-turn dialogue data set, as follows: (1) repeated answer, which means that no duplicate answers should appear in the responses generated by LLMs; (2) anaphora matching, which involves correctly identifying and matching the abbreviations or aliases of medical professional terms used in the dialogue; and (3) key information, where LLMs can recognize and include all relevant information from the question in their response, particularly those that have been repeated 2 or more times in the questions. The content performance criteria used for scoring are outlined in Table 2.

**Table 2.** Summary of evaluation aspects, indicators, criteria, and data sets.

Evaluation aspects		Data sets	Evaluation criteria	Score
**Medical professional capabilities**		Both		
	Accuracy^a^		No medical knowledge errors are present in the answer	1
	Informativeness		Comprehensive: answers include additional information beyond the expectations	2
	Expansiveness		Answers include content from aspects other than medical knowledge included in the question	1
	Logic^a^		On topic: the answers address the topic directly and positively	2
	Prohibitiveness		The model can correctly identify medical or prohibited terms	1
	Sensitivity		There is no political sensitivity expressed in the answers of chatbots by LLM^b^	1
**Social comprehensive capabilities**		Both		
	Comprehension		Understandable: the answers are intuitive and easy to understand	1
	Tone		The answers use correct modal particles and symbols	1
	Empathy		The answers can accurately empathize with the patient	1
	Social decorum		Appropriate: matching 3 or more keywords	2
**Contextual capabilities**		Multiple-turn dialogue		
	Repeated answer		The model has no duplicate answers	1
	Anaphora matching		The model can identify medical professional abbreviations and aliases	1
	Key information		The model can identify key information that appears 2 or more times	1

a Highest priority. If the score of an item is 0, no further evaluation was conducted on either medical professional capability.

b LLM: large language model.

#### Computational Robustness

To evaluate the robustness of the LLMs, 5 extended data sets were created based on first-round questions in the multiple-turn dialogue data set described above. Specifically, the following strategies were used to rephrase each original question and create 10 rephrasing questions: (1) rephrasing the question or sentence but maintaining the semantics (data set A), (2) rephrasing the question or sentence and changing the semantics (data set B), (3) rephrasing the question or sentence by introducing punctuation errors (data set C), (4) rephrasing the question or sentence by introducing grammatical errors (data set D), and (5) rephrasing the question or sentence by introducing spelling errors (data set E). Data sets A-E were used to evaluate the robustness of the LLMs from different common scenarios, which could be classified into 3 anomaly categories. Specifically, data set A was used for the adversarial success rate, data set B for the noise success rate, and data set C-E for the input error success rate.

For each data set, the original and rephrased questions were inputted into the LLMs, and 3 metrics were calculated according to the LLMs’ answers as follows [16,17]: (1) the semantic consistency rate (*R*_1_) represents the proportion of the answer able to maintain the same semantics when inputting a rephrasing question, (2) the semantically inconsistent but medically sound rate (*R*_2_) means that the semantics of the answer has changed but is medically sound when inputting the rephrased question, and (3) the complete error rate (*R*_3_) means that the semantics of the answer have changed and that there is a medical error when inputting a rephrasing question.

### Medical Data Sets in Chinese

Two medical data sets in Chinese were created: medical multiple-turn dialogues and case reports. The data sets [25] include a total of 34 cases, with 27 cases for multiple-turn dialogue and 7 case reports. Data sets included medical scenarios, questions, suspected diagnoses given by LLMs, disease types, and classification of medical questions. The medical questions were sorted into 6 categories: clinical manifestations, treatment, ancillary tests, lifestyle habits, etiology, and prognosis. Most questions focused on patients’ self-reported symptoms and their treatments. The data sets contain 14 types of diseases: systemic diseases, digestive system diseases, brain diseases, heart diseases, bone diseases, chest diseases, vascular diseases, eye diseases, uterine diseases, urinary system diseases, nasopharyngeal diseases, oral diseases, skin diseases, and accidental injuries. Some specific common diseases featured in the data sets are metabolic diseases like diabetes mellitus, gastrointestinal diseases such as gastritis and hyperacidity, and critical diseases like Parkinson disease and heart failure.

### Benchmarks Based on ChatGPT, ERNIE Bot, and Dr PJ

#### Analysis of the Results in Two Medical Scenarios

As shown in Table 3, three assessment aspects were covered in the multiple-turn dialogue evaluation: medical professional capabilities, social comprehensive capabilities, and contextual capabilities. Table 3 shows the total scores of each assessment and the scores of specific indicators. Dr PJ outperformed ChatGPT and ERNIE Bot, with total scores of 13.95, 13.41, and 12.56, respectively. ChatGPT achieved a slightly higher score of 6.30 in medical professional capabilities, compared to 6.25 for Dr PJ and 5.63 for ERNIE Bot. Although ChatGPT performed better in the assessment of medical professional capabilities, Dr PJ had a higher score for accuracy, meaning that the answers were harmless and that Dr PJ performed better in the evaluation of safety. As for social comprehensive capabilities, ChatGPT, ERNIE, and Dr PJ achieved scores of 4.26, 4.33, and 4.70, respectively. Dr PJ achieved a score of 3.00 for context relevance, while ChatGPT and ERNIE Bot achieved scores of 2.85 and 2.59, respectively.

As shown in Table 4, two assessment aspects were covered in the case report evaluation: medical professional capabilities and social comprehensive capabilities. Dr PJ outperformed ChatGPT and ERNIE Bot, with total scores of 10.14, 8.71, and 8.00, respectively. As for medical professional capabilities, Dr PJ achieved 6.86, higher than that of ChatGPT (6.43) and ERNIE Bot (5.71). Similarly, Dr PJ had the highest score (1.00) for accuracy in the evaluation of medical professional capabilities. In addition, Dr PJ had the same scores as ChatGPT regarding informativeness and expansiveness. As for social comprehensive capabilities, the scores for Dr PJ, ChatGPT, and ERNIE Bot were 3.29, 2.29, and 2.29, respectively. Specific scores for each indicator can be found in Table 4.

**Table 3.** The content performances of chatbots in medical scenarios on multiple-turn dialogues.

Evaluation indicators		Chatbots		
		ChatGPT	ERNIE Bot	Doctor PuJiang
Total score (maximum score: 16)		13.41	12.56	13.95
**Medical professional capabilities (maximum score: 8)**		6.30	5.63	6.25
	Accuracy	0.91	0.79	0.94
	Informativeness	1.40	1.22	1.31
	Expansiveness	0.19	0.12	0.17
	Logic	1.81	1.50	1.84
	Prohibitiveness	1.00	1.00	1.00
	Sensitivity	1.00	1.00	1.00
**Social comprehensive capabilities (maximum score: 5)**		4.26	4.33	4.70
	Comprehension	0.96	0.96	0.96
	Tone	0.96	1.00	1.00
	Empathy	0.70	0.70	0.85
	Social decorum	1.63	1.67	1.89
**Contextual capabilities (maximum score: 3)**		2.85	2.59	3.00
	Repeated answer	0.96	0.81	1.00
	Anaphora matching	0.96	0.85	1.00
	Key information	0.93	0.93	1.00

**Table 4.** The content performances of chatbots in medical scenarios with the case report.

Evaluation indicators		Chatbots		
		ChatGPT	ERNIE bot	Doctor PuJiang
Total score (maximum score: 13)		8.71	8.00	10.14
**Medical professional capabilities (maximum score: 8)**		6.43	5.71	6.86
	Accuracy	0.86	0.71	1.00
	Informativeness	1.43	1.14	1.43
	Expansiveness	0.43	0.43	0.43
	Logic	1.71	1.43	2.00
	Prohibitiveness	1.00	1.00	1.00
	Sensitivity	1.00	1.00	1.00
**Social comprehensive capabilities (maximum score: 5)**		2.29	2.29	3.29
	Comprehension	1.00	1.00	1.00
	Tone	0.29	0.14	0.71
	Empathy	0.00	0.14	0.29
	Social decorum	1.00	1.00	1.29

#### Comprehensive Review of Detailed Case Reports

The comments of 2 case reports by 5 medical experts are shown in Multimedia Appendix 1. Overall, all 3 LLMs performed well in correctly understanding patients’ questions. They could comprehend the questions asked by patients and respond with logical answers. However, Dr PJ outperformed the others in terms of sociality. Additionally, Dr PJ answered the questions in an orderly manner, with clear and intuitive serial numbers listed.

#### Computational Robustness Performance

The results in Table 5 show that Dr PJ outperformed ChatGPT and ERNIE Bot in the semantic consistency rate, with a higher adversarial success rate, noise success rate, and input error success rate. This indicates that Dr PJ was the best at maintaining the same semantics of the model answers when questions were paraphrased. Furthermore, in the complete error rate category, both Dr PJ and ERNIE Bot had lower error rates than ChatGPT, suggesting that the semantics of the answer changed when the question was altered. Dr PJ also had a low probability of medical errors.

**Table 5.** The robustness of 3 chatbots for the medical consultation detailed answer task.

Chatbots, anomaly category, and data set			*R*_1_^a^ (%)	*R*_2_^b^ (%)	*R*_3_^c^ (%)
**ChatGPT**					
	**ASR^d^**				
		Data set A	15	65	20
	**NSR^e^**				
		Data set B	15	55	30
	**IESR^f^**				
		Data set C	0	100	0
		Data set D	30	40	30
		Data set E	20	80	0
**ERNIE Bot**					
	**ASR**				
		Data set A	10	85	5
	**NSR**				
		Data set B	0	100	0
	**IESR**				
		Data set C	0	100	0
		Data set D	20	80	0
		Data set E	20	80	0
**Doctor PuJiang**					
	**ASR**				
		Data set A	15	80	5
	**NSR**				
		Data set B	35	65	0
	**IESR**				
		Data set C	60	40	0
		Data set D	50	40	10
		Data set E	80	20	0

a *R*_1_: semantic consistency rate.

b *R*_2_: semantically inconsistent but medically sound.

c *R*_3_: complete error rate.

d ASR: adversarial success rate.

e NSR: noise success rate.

f IESR: input error success rate.

## Discussion

### Principal Findings

In this study, we introduced a set of comprehensive evaluation criteria for assessing LLMs’ performances in medical contexts, considering aspects such as medical professional capabilities, social comprehensive capabilities, contextual capabilities, and computational robustness. We compared ChatGPT and ERNIE Bot with Dr PJ in 2 medical scenarios: multi-turn dialogues and case reports. Experimental results show that Dr PJ outperformed ChatGPT and ERNIE Bot in handling various forms of the same question in these 2 scenarios.

Recently, LLMs have achieved rapid advancements and demonstrated technical potential. However, only a few question-and-answer evaluation methods have been developed for nonmedical fields or accuracy aspects. Liu et al [26] presented a research summary for ChatGPT/GPT-4 suggesting that there are several evaluation aspects to consider, such as engineering performance, scenario, user feedback, and negative impacts. Similarly, West [17] evaluated the accuracy of ChatGPT-3.5 and ChatGPT-4 in answering conceptual physics questions by assessing correctness, confidence, error type, and stability. Further, Tan et al [16] compared responses from 6 English and 2 multilingual data sets, totaling 190,000 cases, and they discovered that ChatGPT outperformed similar models in most results but struggled with questions requiring numerical or time-based answers. However, the team’s evaluation metrics such as the minimal functionality test, invariance test, and directional expectation test [16] are primarily focused on model performances and stability. Unlike general question-answering domains, medical data sets require a more comprehensive evaluation approach. It is essential to not only focus on the LLMs’ performances but also consider the physical and psychological state of the questioner, as well as potential patients seeking medical assistance from a medical professional’s perspective. As a result, we propose content evaluation criteria including both medical and social capabilities. Simultaneously, in a recent publication comparing physicians versus LLMs’ responses to patient questions, the researchers assessed the quality of information and empathy of the responses on a 5-point scale [27]. Moreover, a recent study on radiation oncology physics showed that GPT-4 performed better in answering highly specialized radiation oncology physics questions after labeling. However, results were obtained where human expertise won out, suggesting the importance of the diversity of expertise and contextual inference capabilities [13]. Correspondingly, contextual capabilities are incorporated as a crucial component to evaluate LLMs’ contextual inference professionally and objectively. We believe that the comprehensiveness of Chinese data sets is equally important. For example, our latest proposed medical data sets in Chinese include common and critical diseases from 14 different clinical departments. Furthermore, our open-source data sets can facilitate a fairer evaluation process and expedite the global assessment and advancement of LLMs applied to medical data sets in Chinese.

Many current models are data hungry and necessitate labor-intensive labeling [28]. The advent of medical knowledge graphs and foundation models, which enable training without labeled data and professional medical knowledge, has driven the application of AI throughout the clinical workflow, including triage, diagnosis, and clinical management [4,29,30]. Inspired by these advancements, we developed Dr PJ, an LLM based on massive medical data sets in Chinese. Given the highly specialized nature of medical care, training LLMs in this field requires strict supervision to ensure medical professionalism. Simultaneously, humanistic care, a fundamental aspect of doctor-patient communication, is crucial for human-computer interaction [31]. Unlike ChatGPT and ERNIE Bot, which are general AI models pretrained on general internet data, Dr PJ was built for medical applications and has been trained using medical texts. When applying these models to multiple-turn dialogues, our model achieved the highest total score. This result shows that the higher medical expertise score of ChatGPT resulted from informativeness and expansiveness, while our model achieved better accuracy and medical safety. Additionally, we evaluated the robustness of models by changing the method of inputs or the order of words. In the real world, patients may enter their symptoms in different ways or may remember diseases or drugs incorrectly. The word order may also influence the natural language understanding [32]. Therefore, it is important to measure the robustness of medical models to deal with various inputs. Dr PJ had higher semantic consistency and a lower complete error rate compared to ChatGPT, indicating better robustness. Although the developers of OpenAI believe that ChatGPT performs well in translation, it does not perform stably in different modes of questioning. This indicates that the language barrier in foundation models is an important factor to consider.

### Limitations

Limitations remain in the evaluation system and LLM development. First, the evaluation criteria primarily rely on subjective scoring by a group of medical professionals. Although this approach aligns with the principles of the medical domain, it can introduce a certain bias into the results, and the human-scoring system can waste time and human resources. Second, our data set mainly focuses on Chinese medicine, which has language and cultural limitations. This may have some impact on the generalizability of the findings. Expanding the scope of the data set in future studies would be a worthwhile research direction to enhance the reliability and generalizability of the study.

### Future Directions

To improve evaluation efficiency and reduce bias, future work on the combination of automated model evaluation is needed. Moreover, the scale of medical data sets for evaluation is still limited, so we encourage research collaborations to help expand the current evaluation data set with more Chinese medical data sets to construct a more comprehensive evaluation data set. In addition, foundation models with a greater number of parameters have the potential to yield better accuracy. We can also potentially enhance the model performance by training the model with more complex parameters. Finally, note that using different prompts may have an impact on model output [33]. Therefore, evaluations of different prompting strategies for models should be conducted to select those suitable for medical scenarios.

### Conclusions

This work proposed an assessment system, composed of a set of evaluation criteria, open-source medical data sets in Chinese, and a benchmark of 3 chatbots. Medical experts evaluated the LLMs and found that 3 chatbots (ChatGPT, ERNIE Bot, and Dr PJ) could understand patients’ questions and provide logical answers. Through a comparison using the proposed evaluation criteria, we found that Dr PJ outperformed the other 2 models with more accurate medical knowledge and humanistic care. Overall, the study results underscore the need for continuous research and development in LLMs to ensure their safe and effective use in medical scenarios.

## Supplementary material

10.2196/57674 Multimedia Appendix 1 Comments of detailed case reports.

## Abbreviations

AI: artificial intelligence
Dr PJ: Doctor PuJiang
LLM: large language model
