# Supplementary material for: Data Set and Benchmark (MedGPTEval) to Evaluate Responses From Large Language Models in Medicine: Evaluation Development and Validation
Source: JMIR Med Inform. 2024 Jun 28;12:e57674. doi: 10.2196/57674 (PMC11225096; doi:10.2196/57674)
Supplement: Multimedia Appendix 1 [file medinform-v12-e57674-s001.doc]

**Appendix 1**

Table 1 Comments of detailed case reports.

| Case report |  | Chatbots | | |
| --- | --- | --- | --- | --- |
|  |  | ChatGPT | ERNIE Bot | Dr. PJ |
| No.2 | Answer | First, chronic atrophic gastritis is not necessarily a precancerous condition, but it does represent a high-risk factor for gastric cancer. In your case, it is recommended that you consult with your physician as soon as possible to determine if you need further testing or treatment.  The following are answers to your questions:  For patients with chronic gastritis, it is recommended to control the intake of red meat, but not completely. Moderate consumption of lean beef or lean lamb is possible, but high-fat and greasy meats such as fatty beef and fatty lamb should be avoided.  Coffee stimulates gastric acid secretion and is not very friendly to people with chronic gastritis, but it is not completely unacceptable. It is recommended to drink it in moderation, preferably with some milk or sugar to neutralize the stimulation of stomach acid.  Whether or not you need medication depends on your condition. If your symptoms are mild, you may be able to relieve them by making changes to your diet and lifestyle. If your symptoms are severe, your doctor may recommend medication such as an acid suppressant to reduce your symptoms.  The exact timing of your review should depend on your condition and your doctor's recommendations. In general, a review every 6 months to 1 year is recommended to see how your condition is changing. If your symptoms worsen or new symptoms appear, consult your doctor promptly. | First, chronic atrophic gastritis is a common stomach disease, but it is not necessarily a precancerous lesion, so there is no need to be overly nervous. According to your description, your symptoms are not serious, and you have taken measures to control your diet.  Regarding your diet, you are advised to pay attention to control your diet in your daily life and avoid excessive intake of foods high in fat, sugar, and salt, as well as too much caffeine. You can choose foods that are low in fat, high in fiber and high in protein, such as lean meat, fish, beans, vegetables, and fruits.  As for medications, you are advised to consult your doctor for advice. In general, chronic atrophic gastritis does not require special treatment, but if the symptoms are severe or there are other uncomfortable symptoms, you may consider using some medications to relieve the symptoms. Commonly used medications include antacids, gastric mucosal protectors, and gastric motility drugs.  Regarding the timing of review, it is recommended that you have a gastroscopy every 3 to 6 months after your symptoms have subsided so that any potential problems can be detected and addressed promptly. If symptoms persist or worsen, it is recommended that you undergo further examination and treatment under the guidance of your doctor. | Hi, I can understand the anxiety you are feeling right now when I hear your concerns and questions. I will do my best to answer your questions and offer some suggestions to help relieve your symptoms and manage your progress.  First, to answer your questions:  1. You can eat high protein foods such as beef and lamb in moderation when you go out to eat but be careful not to cook them in a way that is too greasy or fried.  2. Avoid or consume small amounts of caffeine-rich beverages such as cola, black tea, and beverages containing large amounts of sugar.  3. The use of medications for the treatment of chronic atrophic gastritis or other diseases is not recommended without the advice of a physician, as different patients require individualized medication regimens for different conditions.  4. Periodic review is recommended to monitor disease progression and to adjust the time interval and regimen selection of the treatment plan. Usually, depending on the patient's specific situation, a checkup once every 6 months to about 1 year is usually sufficient.  In addition, besides paying attention to your dietary habits, it is also very important for you to maintain a good routine and exercise appropriately to strengthen your immune system and other aspects of self-care measures. It is also recommended that you seek the help and advice of a medical professional to better understand the changes in your health status and take appropriate prevention and intervention strategies. |
|  | Comments | The answers were provided with detailed explanations, combined with the suggested medication and recommendations on patient follow-up, and each of the questions was answered one by one. Generally, the responses were moderate and complete. | The answers were provided with detailed explanations. All questions were answered. Medication and patient follow-up related information were provided. Generally, the responses were moderate and complete. | The answers were provided with detailed explanations, combined with the suggested medication and recommendations on patient follow-up. Each of the questions was answered orderly with serial numbers listed, which was more intuitive. In addition, humanistic concern was embodied in the expression above, complying with the patient's medical needs. |
| No.4 | Answer | Based on the diagnosis of a T value of -3.5, your mother's degree of osteoporosis is considered severe and requires measures for treatment and prevention. Osteoporosis is a chronic disease that, if left untreated and uncontrolled, can lead to serious consequences such as osteoporotic fractures.  Aspects of treatment for osteoporosis include medication, nutritional supplements, exercise, and precautions for daily living. Your doctor may advise your mother to take medication, such as calcium supplements, vitamin D, estrogen replacement therapy, and bone resorption inhibitors. It is also important to take nutritional supplements, including increasing the intake of calcium, vitamin D, and protein, as well as limiting alcohol consumption and quitting smoking. Exercise can increase bone density and prevent osteoporosis, and your doctor may recommend some aerobic exercise and gravity training in moderation.  Injectable therapy may refer to bone density enhancing medications such as sodium succinate and omeprazole, which need to be injected once or several times a year. These medications do not require hospitalization and can be administered on an outpatient basis or in a day surgery, but the exact cost will depend on the area and hospital.  Osteoporosis has a greater impact on life and can easily lead to fractures and shortening of height, as well as affecting the quality of life. Therefore, it is very important to treat and control osteoporosis early.  Regarding lifestyle habits, it is recommended that your mother increase exercise and moderate intake of calcium and vitamin D, while avoiding bad habits such as smoking and alcohol consumption. Yoga is a light exercise that can be performed, but excessive twisting and bending needs to be avoided to avoid problems such as fractures. It is recommended that your mother consult a doctor or professional yoga teacher to develop an exercise program that suits her needs. | Based on the information you provided, your mother's osteoporosis symptoms may be severe and require formalized treatment. The following is my response:  Based on the information you provided, your mother's T-value of -3.5 and Z-value of -2.1 indicate that she has low bone density, which may lead to an increased risk of fracture. Therefore, her condition may be more severe.  Formalized treatment usually includes the following:  Medications: These include calcium and vitamin D supplements, calcitonin, estrogen, and other medications that can help increase bone density and reduce the risk of fracture.  Dietary modifications: Add calcium and vitamin D-rich foods to your diet, such as milk, fish, and beans.  Exercise: Proper exercise can help increase bone density and bone mass.  Lifestyle changes: Reduce bad habits such as smoking and alcohol consumption and maintain proper weight and diet.  The medication to be injected may be calcitonin or estrogenic drugs, which need to be injected according to the doctor's recommendation. These medications usually require inpatient injections, and the cost may vary by region and hospital.  Osteoporosis may have a greater impact on life as it can lead to fractures, physical pain, and mobility problems. Your mother is advised to take care of herself in her daily life to avoid falls and other accidental injuries.  Your mother is advised to increase her diet with foods rich in calcium and vitamin D, such as milk, fish, and beans. Proper exercise can help increase bone density and bone mass. She is also advised to reduce bad habits such as smoking and alcohol consumption and maintain a proper weight and diet. If she has other health problems, such as high blood pressure and diabetes, she should also consult her doctor promptly. | Hi, I understand the concerns you and your mother are having. I will try to answer your questions and provide some help and advice:  1. Based on the information you have provided; it appears that your mother has been diagnosed with osteoporosis (osteopenia) and her T value is -3.5 and Z value -2.1 indicating that she is already in a moderate to severe state of reduced bone density. This is a common bone disease that is often associated with age, gender, and other factors. If left untreated and unmanaged it may lead to an increased risk of complications such as fractures. Therefore, timely treatment and management is needed to control progression and reduce risk.  2. The aspects of formalized treatment include both pharmacological and non-pharmacological interventions. Non-pharmacological treatments include exercise (e.g., walking or yoga) to strengthen muscles and improve balance, and a balanced diet and avoidance of smoking and alcohol. The specific treatment method should be determined by the patient's specific situation and under the guidance of a professional doctor.  3. I am not sure about the specific name of the drug, but, the injectable drugs for osteoporosis are mainly bisphosphonate preparations such as alendronate or other similar substances. These medications can help prevent fractures by strengthening bones and promoting new bone formation. The need for hospitalization depends on the circumstances and the medication regimen and may require hospitalization if long-term observation and monitoring is required. Costs will vary depending on the case, so you can check with your local health care provider for details.  The impact on daily life varies from person to person. Some people may not be affected as much, but others may have pain or other symptoms that affect their daily activities and work. For older people like your mother, it is very important to maintain a good lifestyle, such as maintaining a moderate amount of physical activity and a proper dietary structure to help maintain good health. It is also important to follow the doctor's instructions for regular check-ups to monitor the effectiveness of the treatment and adjust the treatment plan.  Finally, I would like to remind you that the above is only general advice and information, if you have any questions or uncertainties, it is best to consult a professional physician for more accurate advice and solutions. I hope my reply will be helpful to you! |
|  | Comments | Each of the questions was well-answered with detailed clarification including advice on both treatment plans and lifestyle habits. Answers to yoga related question was elaborated in this answer as well. | Each of the questions was well-answered but with fewer details. Both treatment and lifestyle habits recommendations were provided. Overall, the responses were moderate. | Each of the questions was well-answered with detailed clarification including advice on both treatment plans and lifestyle habits. A clear serial number on each question correspondingly. Both the correctness and humanistic concern of the responses were satisfactory. |
